# A nomogram prediction model for the TP53mut subtype in endometrial cancer based on preoperative noninvasive parameters

**DOI:** 10.1186/s12885-023-11234-1

**Published:** 2023-08-01

**Authors:** Wei Wang, Xiaoting Li, Yunong Gao, Hong Zheng, Min Gao

**Affiliations:** 1 Department of Gynecologic Oncology, Key Laboratory of Carcinogenesis and Translational Research (Ministry of Education/Beijing), Peking University Cancer Hospital & Institute, Hai Dian District, Beijing, 100142 China; 2 Department of Radiology, Key Laboratory of Carcinogenesis and Translational Research (Ministry of Education/Beijing), Peking University Cancer Hospital & Institute, Hai Dian District, Beijing, 100142 China

**Keywords:** Molecular subtypes, Endometrial carcinoma, Next-generation sequencing (NGS), Nomogram, Prediction

## Abstract

**Background:**

The molecular subtypes of endometrial carcinoma are significantly correlated with survival outcomes and can guide surgical methods and postoperative adjuvant therapy. Among them, the TP53mut subtype has the worst prognosis and can only be determined by detection after surgery. Therefore, identifying preoperative noninvasive clinical parameters for early prediction of the TP53mut subtype would provide important guidance in choosing the appropriate surgical method and early warning for clinicians. Our study aimed to establish a model for the early prediction of the TP53mut subtype by using preoperative noninvasive parameters of endometrial cancer and screen out potential TP53mut patients.

**Methods:**

Information and pathological specimens of 376 patients who underwent surgery for FIGO stage I-IV endometrial cancer in the Department of Gynecology, Peking University Cancer Hospital, from June 2011 to July 2020 were collected, and 178 cases were finally included in the study as the training dataset (part A). Thirty-six cases from January 2022 to March 2023 were collected as the validation dataset (part B). Molecular subtyping was performed using a one-stop next-generation sequencing (NGS) approach. Compared with the TP53mut subtype, the POLE EDM, MSI-H and TP53 wild-type subtypes were defined as non-TP53mut subtypes. Univariate Cox regression analysis and multivariate logistic analysis were performed to determine the preoperative clinical parameters associated with the TP53mut subtype. A nomogram prediction model was established using preoperative noninvasive parameters, and its efficacy in predicting TP53mut subtype and survival outcomes was verified.

**Results:**

The TP53mut subtype was identified in 12.4% of the part A and 13.9% of the part B. Multivariate logistic regression analysis showed that HDL-C/LDL-C level, CA125 level, and cervical or lower uterine involvement were independent influencing factors associated with the TP53mut subtype (*p* = 0.016, 0.047, <0.001). A TP53mut prognostic model (TPMM) was constructed based on the factors identified in the multivariate analysis, namely, TPMM = -1.385 × HDL-C/LDL-C + 1.068 × CA125 + 1.89 × CI or LUI, with an AUC = 0.768 (95% CI, 0.642 to 0.893) in the part A. The AUC of TPMM for predicting TP53mut subtype in the part B was 0.781(95% CI, 0.581 to 0.980). The progression-free survival (PFS) and overall survival (OS) of patients with the TP53mut subtype were significantly worse than those of patients with the non-TP53mut subtype, as predicted by the model in the part A.

**Conclusions:**

TP53mut prediction model (TPMM) had good diagnostic accuracy, and survival analysis showed the model can identify patients with different prognostic risk.

## Background

Endometrial cancer (EC) is the most common gynecologic malignancy, and the incidence of this disease has been increasing yearly. Globally, the age-standardized incidence of EC increased by 0.69% per year between 1990 and 2019 [1]. In the past, EC was classified into type I and type II. The pathological type referred to as type I EC is mainly endometrioid cancer, which is estrogen dependent. Other pathological types, such as serous carcinoma, clear cell carcinoma and mixed carcinoma, which are non-hormone-dependent and have a poor prognosis, are referred to as type II endometrial carcinoma [2]. However, there is great inconsistency between these classifications and clinical outcomes. To solve this problem, The Cancer Genome Atlas (TCGA) in 2013 defined four EC prognostic subgroups based on the comprehensive genomic characteristics of EC: POLE mutant type, MSI-H type, low-copy type and high-copy type [3]. This molecular classification system is consistent with the clinical outcomes; that is, POLE hypermutant tumors are associated with good progression-free survival, MSI-H and low-copy tumors with moderate progression-free survival, and high-copy tumors with poor progression-free survival. At present, this four-category molecular typing system is considered the gold standard for molecular typing of endometrial carcinoma due to its good correlation with prognosis. However, the TCGA molecular typing system is difficult to widely use in clinical practice due to its complex operation and high testing cost. Thus, scholars have proposed the Proactive Molecular Risk Classifier for Endometrial Cancer (ProMisE) method [4] and TransPORTEC method [5] to improve the TCGA molecular typing system, and the prognostic information obtained is basically consistent with the TCGA molecular typing system; that is, the prognosis associated with POLE hypermutant tumors is the best, that with MSI-H and TP53 wild-type/ no specific molecular profile (NSMP) tumors is intermediate, and that with TP53 mutant tumors is the worst. Although molecular typing has been widely accepted by clinicians in recent years, noninvasive methods were used to predict the endometrial cancer subtypes always by machine learning [6, 7], while there have been few clinical studies on the establishment of models based on preoperative clinical parameters to predict the molecular subtype of EC.

A nomogram is a graphical prediction model that analyzes multiple quantitative and qualitative variables to predict the probability of a specific event. It can be used to illustrate risk in an individual patient. The model is based on logistic regression and Cox regression, and the results are presented visually. The scoring criteria are formulated according to the regression coefficient of the model, and each independent variable is assigned a score. Then, the probability of an end event in each patient is calculated by the conversion function. Nomogram prediction models have been used to predict tumor recurrence, metastasis and survival outcomes in several carcinomas [8–11], but few models have been reported for EC risk prediction.

This study aimed to develop a nomogram prediction model based on a one-stop next-generation sequencing (NGS) approach by utilizing preoperative clinical parameters to predict the TP53mut subtype, which has the worst clinical outcomes, and provide disease status information before surgery to help clinicians develop appropriate surgical methods and postoperative adjuvant therapy.

## Methods

### Study population and data collection

**Fig. 1:**
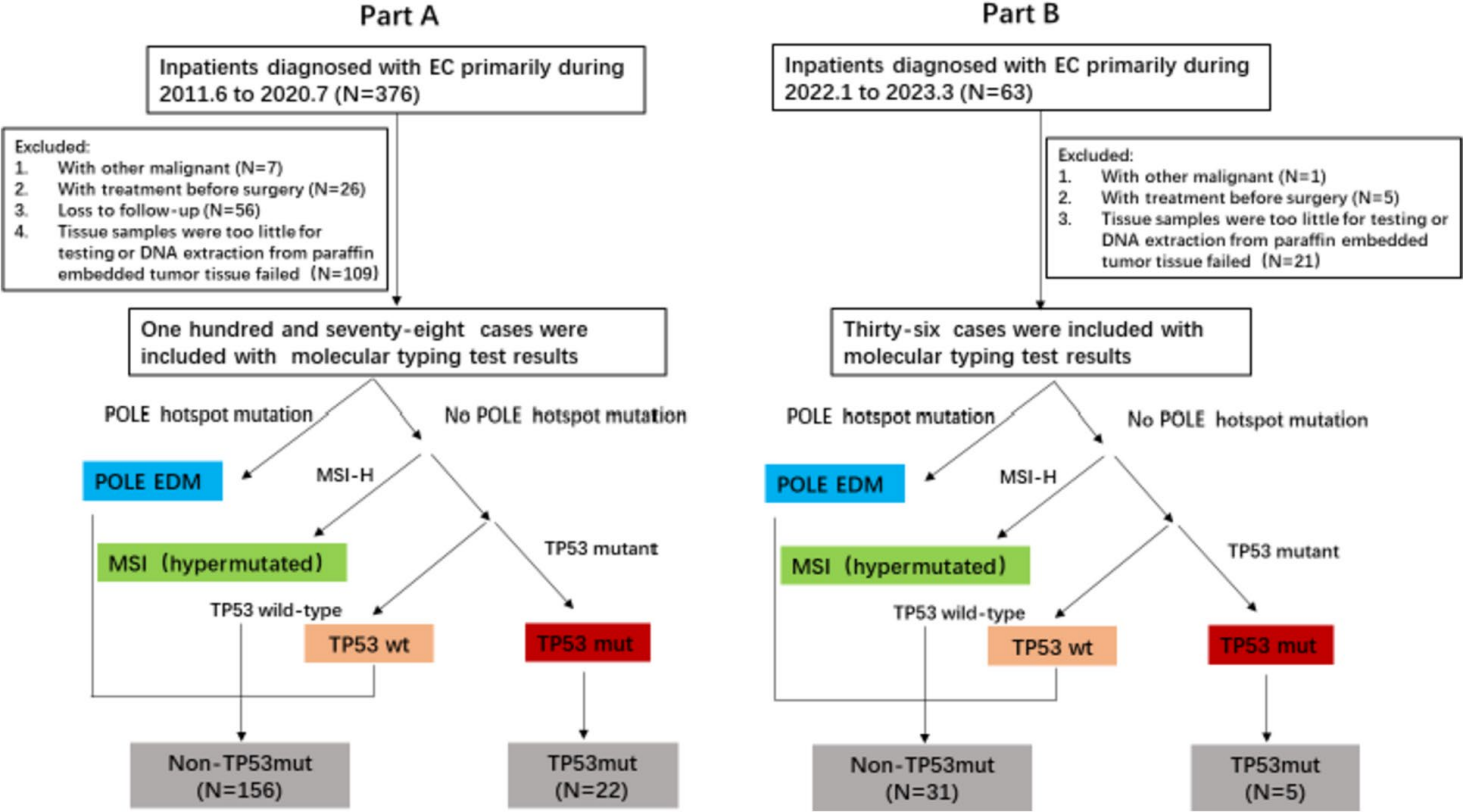
Patient enrollment process and one-stop NGS molecular typing procedure

The study was approved by the Ethics Review Committee of Beijing Cancer Hospital with reference number 2021YJZ21 and was conducted in accordance with the guidelines of the Declaration of Helsinki. All participants provided written informed consent at the time of sample collection. This study included two part of patients: part A (training dataset) for exploring the preoperative predictors and establishing a model to predict TP53mut subgroup; part B (validation dataset) for validating the model yielded from part A. Part A: Information and pathological specimens of 376 patients who underwent surgery for FIGO stage I-IV EC in the Department of Gynecology, Peking University Cancer Hospital, from June 2011 to July 2020 were collected, and 178 cases were finally included. Part B: Information and pathological specimens of 63 patients in our center from January 2022 to March 2023 were collected, and 36 cases were finally included. The patient enrollment process was shown in Fig. 1.

The standard scope of operation included total hysterectomy and bilateral salpingo-oophorectomy. Lymph node resection was performed in selected patients. None of the patients received preoperative chemoradiotherapy or hormone therapy. Two gynecological pathologists examined the pathological specimens. A 1 mm diameter surgical tumor specimen was provided, and at least 15 slides were provided for sequencing. Adjuvant therapy was tailored according to the surgical stage and histological type. Patients with high-risk endometrioid cancer were given vaginal brachytherapy or whole-pelvic radiotherapy. Vaginal brachytherapy was mainly limited to patients undergoing surgical lymph node evaluation. Patients with non-endometrioid cancer or advanced endometrioid cancer were treated with a combination of chemotherapy and radiation. Paclitaxel combined with carboplatin was the standard chemotherapy regimen.

Preoperative general characteristics, hematological and imaging examination results, pathological characteristics, postoperative adjuvant therapy results, and survival follow-up results were collected. Neutrophils, monocytes, lymphocytes, platelets and hemoglobin levels were counted as the mean ± SD × 10^9^/L in routine blood tests. The ratio data were grouped by the median, and the blood biochemical results were divided into three categories: below the reference range, within the reference range, and above the reference range. Tumor biomarkers were divided into two categories: within the reference range and above the reference range. The image data were determined by two radiographers with more than 10 years of working experience.

### Molecular classification and grouping

Genomic analysis of tumor tissue was performed using the OncoScreen Plus panel of 520 cancer-related genes. Tissue DNA was extracted from formalin-fixed, paraffin-embedded (FFPE) tumor tissues using the QIAamp DNA FFPE tissue kit (Qiagen, Hilden, Germany) in accordance with the manufacturer's standard protocol (Qiagen, Hilden, Germany). Capture targeted sequencing was performed using 520 gene panels (OncoScreen PlusTM, Burning Rock Biotech, Guangzhou, China) [12–14]. Data analysis included variable invocation and interpretation, copy number changes, tumor mutation burden (TMB) estimation, and microsatellite instability (MSI) status assessment. The aneuploidy score was calculated using the total number of previously reported chromosome arms gained or lost. In this study, only polar hotspot mutations at codons 286, 297, 411, 456 and 459 in the polar exonuclease domain were considered the causal mutations of the supermutation and were defined as the POLE EDM subtype. Patients were divided into four molecular subtypes: POLE EDM, MSI-H, TP53 wild-type and TP53mut. In this study, compared with the TP53mut subtype, the POLE EDM, MSI-H and TP53 wild-type subtypes were defined as non-TP53mut subtypes. The one-stop NGS molecular typing procedure for part A and part B was shown in Fig. 1.

One-stop NGS molecular typing procedure as follows: The panel analyzed the mutant genes existing in patients with endometrial cancer, and classified the patients one by one according to the mutation results. First, the tumors were evaluated for polymerase-E (POLE) exonuclease domain mutations (EDMs). Second, MSI hypermutation was evaluated. Third, the TP53 mutant subtype and TP53 wild-type subtype were evaluated according to whether the tumor had TP53 mutation. In this study, the TP53mut group and non-TP53mut group (including POLE EDM, MSI hypermutation and TP53 wild type) were reclassified.

### Follow up

All patients were followed up after operation at three months’ interval during the first two years, six months’ interval since the third year, and one year’s interval since the fifth year. The data of disease progression and death were recorded. Progression-free survival was calculated from the date of operation to the data of occurence of disease progression or censored at the date of the last follow up. Overall survival was calculated from the date of operation to the data of death or censored at the date of the last follow up. Progression was defined as local or distant recurrence or death.

### Statistical methods

All statistical analyses were performed using R software (version 4.1.2) and SPSS (version 22.0). The chi-square test or Fisher's exact test was used to compare the differences in categorical variables between the TP53mut and non-TP53mut groups, and the dependent t test or Mann‒Whitney test was used to compare continuous variables. Multivariate logistic regression analysis was used to screen for independent factors for identifying TP53mut patients, a predictive model was established, and a nomogram was generated accordingly. A receiver operating characteristic (ROC) curve was produced, and the area under the curve (AUC), sensitivity, and specificity were calculated. Kaplan‒Meier curves with the log-rank test were used to compare progression-free survival and overall survival between the model-predicted TP53mut group and the model-predicted non-TP53mut group. A two-sided *p* < 0.05 was considered statistically significant.

## Results

### Baseline characteristics of EC patients

In the part A, 178 patients with initial EC treatment were included. There were 22 patients (12.4%) with TP53mut and 156 patients (87.6%) with non-TP53mut. In the part B, 36 patients were included. There were 5 patients (13.9%) with TP53mut and 31 patients (86.1%) with non-TP53mut. Except patients’ age, there were no statistically significant difference in other baseline characters between part A and part B patients. The general conditions of the patients are shown in Table 1.

**Table 1:** Patient characteristics between part A and part B

	**Part A** ***N* = 178**	**Part B** ***N* = 36**	***p***
**Age (years)**			< 0.001
**≤ 50**	90(50.6)	5(13.9)	
**> 50**	88(49.4)	31(86.1)	
**BMI (kg/m)**^**2**^	26.26 ± 4.26	26.78 ± 4.01	0.889
**Complication**			0.211
** No**	109(61.2)	18(50.0)	
** Yes**	69(38.8)	18(50.0)	
**Histological grade**			0.117
** G1-G2**	136(76.4)	23(63.9)	
** G3**	42(33.6)	13(36.1)	
**Family history of cancer**			0.314
** No**	142(79.8)	26(72.2)	
** Yes**	36(202)	10(27.8)	
**Previous cancer history**			0.695
** No**	167(93.8)	35(97.2)	
** Yes**	11(6.2)	1(2.8)	
**Histological subtype**			0.415
** Non-endometrioid carcinoma**	21(11.8)	6(16.7)	
** Endometrioid carcinoma**	157(88.2)	30(83.3)	
**FIGO stage**			0.099
** I**	137(77.0)	23(61.1)	
** II-IV**	41(23.0)	13(38.9)	
**TP53**			0.785
** Non-TP53mut**	156(87.6)	31(86.1)	
** TP53mut**	22(12.4)	5(13.9)	

### Comparisons of preoperative parameters between non-TP53mut and TP53mut patients in the part A

The results showed that the TP53mut group had a higher proportion of internal diseases, namely, hypertension or diabetes (*p* = 0.011), non-endometrioid cancer (*p* = 0.006) and higher FIGO stage (*p* < 0.001). The neutrophil, monocyte and platelet counts in patients with TP53mut were significantly higher than those in patients with non-TP53mut (*p* = 0.007, 0.025, < 0.001). The LDL-C level was higher than the reference range in many TP53mut patients, and the difference was statistically significant (*p* = 0.005). The proportion of HDL-C/LDL-C in TP53mut patients was higher than the median of 0.5, and the difference was statistically significant (*p* = 0.025). The level of CA125 was higher in TP53mut patients than in those without TP53mut (*p* = 0.003). Additionally, more TP53mut patients had cervical involvement or lower uterine involvement according to preoperative imaging (*p* < 0.001; Table 2).

**Table 2:** Comparisons of preoperative parameters between Non-TP53mut and TP53mut patients in the part A

	**Non-TP53mut** ***N* = 156 (%)**	**TP53mut** ***N* = 22 (%)**	***p***
**Age (years)**			0.609
≤ **50**	80(51.3)	10(45.5)	
> **50**	76(48.7)	12(54.5)	
**BMI (kg/m**^**2**^**)**	26.31 ± 4.38	25.88 ± 3.39	0.655
**Complication**			0.011
** No**	101(64.7)	8(36.4)	
** Yes**	55(35.3)	14(63.6)	
**Histological grade**			0.332
** G1-G2**	121(77.6)	15(68.2)	
** G3**	35(22.4)	7(31.8)	
**Family history of cancer**			0.778
** No**	125(80.1)	17(77.3)	
** Yes**	31(19.9)	5(22.7)	
**Previous cancer history**			0.629
** No**	147(94.2)	20(90.9)	
** Yes**	9(5.8)	2(9.1)	
**Histological subtype**			0.006
** Non-endometrioid carcinoma**	14(9.0)	7(31.8)	
** Endometrioid carcinoma**	142(91.0)	15(68.2)	
**FIGO stage**			< 0.001
** I**	137(87.8)	0(0)	
** II-IV**	19(12.2)	22(100)	
** Neutrophils (mean ± SD, × 10**^**9**^**/L)**	3.19 ± 0.98	3.97 ± 1.31	0.007
** Monocytes (mean ± SD, × 10**^**9**^**/L)**	0.32 ± 0.10	0.50 ± 0.68	0.025
** Lymphocytes (mean ± SD, × 10**^**9**^**/L)**	1.65 ± 0.26	1.76 ± 0.51	0.611
** Platelets (mean ± SD, × 10**^**9**^**/L)**	204.3 ± 39.37	242.7 ± 53.12	< 0.001
** Hemoglobin (mean ± SD, g/L)**	128.8 ± 13.48	128.5 ± 11.50	0.627
**Neutrophil/lymphocyte**			0.172
** ≤ 1.96**	81(51.9)	8(36.4)	
** > 1.96**	75(48.1)	14(63.6)	
**Platelet/lymphocyte**			0.097
≤ **142**	106(67.9)	11(50.0)	
** > 142**	50(32.1)	11(50.0)	
**TP (g/L)**			0.193
** Low**	6(3.8)	0(0)	
** Normal**	144(92.4)	22(100)	
** High**	6(3.8)	0(0)	
**Alb (g/L)**			/
** Low**	0(0)	0(0)	
** Normal**	156(100)	22(100)	
** High**	0(0)	0(0)	
**A/G**			> 0.999
** Low**	0(0)	0(0)	
** Normal**	154(98.7)	22(100)	
** High**	2(1.3)	0(0)	
**TCHO (mmol/L)**			0.707
** Low**	1(0.6)	0(0)	
** Normal**	116(74.4)	15(68.2)	
** High**	39(25.0)	7(31.8)	
**TG (mmol/L)**			0.431
** Low**	5(3.2)	0(0)	
** Normal**	99(63.5)	13(59.1)	
** High**	52(33.3)	9(40.9)	
**HDL-C (mmol/L)**			0.862
** Low**	5(3.2)	1(4.5)	
** Normal**	140(89.7)	20(91.0)	
** High**	11(7.1)	1(4.5)	
**LDL-C (mmol/L)**			0.005
** Low**	4(2.6)	0(0)	
** Normal**	124(79.5)	11(50.0)	
** High**	28(17.9)	11(50.0)	
**HDL-C/LDL-C**			0.025
≤ **0.5**	81(51.9)	17(77.3)	
** > 0.5**	75(48.1)	5(22.7)	
**CA125**			0.003
** Normal**	131(84.0)	12(54.5)	
** High**	25(16.0)	10(45.5)	
**CA199**			0.233
** Normal**	96(80.7)	13(68.4)	
** High**	23(19.3)	6(31.6)	
**CEA**			> 0.999
** Normal**	116(98.3)	19(100)	
** High**	2(1.7)	0(0)	
**CI or LUI**			< 0.001
** No**	119(76.3)	7(31.8)	
** Yes**	37(23.7)	15(68.2)	

*Abbreviations*: *TP* Total protein, *Alb* Albumin, *A/G* Albumin/globulin ratio, *TCHO* Total cholesterol, *TG* Triglycerides, *HDL-C* High-density lipoprotein cholesterol, *LDL-C* Low-density lipoprotein cholesterol, *CI or LUI* Cervical involvement or lower uterine involvement according to imaging

### Comparisons of postoperative pathological features between non-TP53mut and TP53mut patients in the part A

Postoperative pathological features included ascites cytology, lymphovascular space invasion (LVSI), deep myometrial infiltration (DMI), and lymph node metastasis (LNM). These pathological factors were dichotomized as yes or no. The univariate analysis of postoperative pathological characteristics showed that there were more patients with positive ascites cytology, DMI, LVSI and LNM in the TP53mut group than in the non-TP53mut group (*p* = 0.019, < 0.001, < 0.001, < 0.001; Table 3).

**Table 3:** Comparisons of postoperative pathological features between Non-TP53mut and TP53mut patients in the part A

	**Non-TP53mut** **N = 156 (%)**	**TP53mut** **N = 22 (%)**	***p***
**Ascites cytology**			0.019
** No**	135(86.5)	14(66.7)	
** Yes**	19(13.5)	7(33.3)	
**DMI**			< 0.001
** No**	128(82.1)	7(31.8)	
** Yes**	28(17.9)	15(68.2)	
**LVSI**			< 0.001
** No**	129(82.7)	4(18.2)	
** Yes**	27(17.3)	18(81.8)	
**LNM**			< 0.001
** No**	156(100)	1(4.5)	
** Yes**	0(0)	21(95.5)	

*Abbreviations*: *DMI* Deep myometrial invasion, *LVSI* Lymphovascular space invasion, *LNM* Lymph node metastasis

### Multivariate logistic analysis of preoperative clinical parameters for predicting TP53mut and nomogram model construction in the part A

**Fig. 2:**
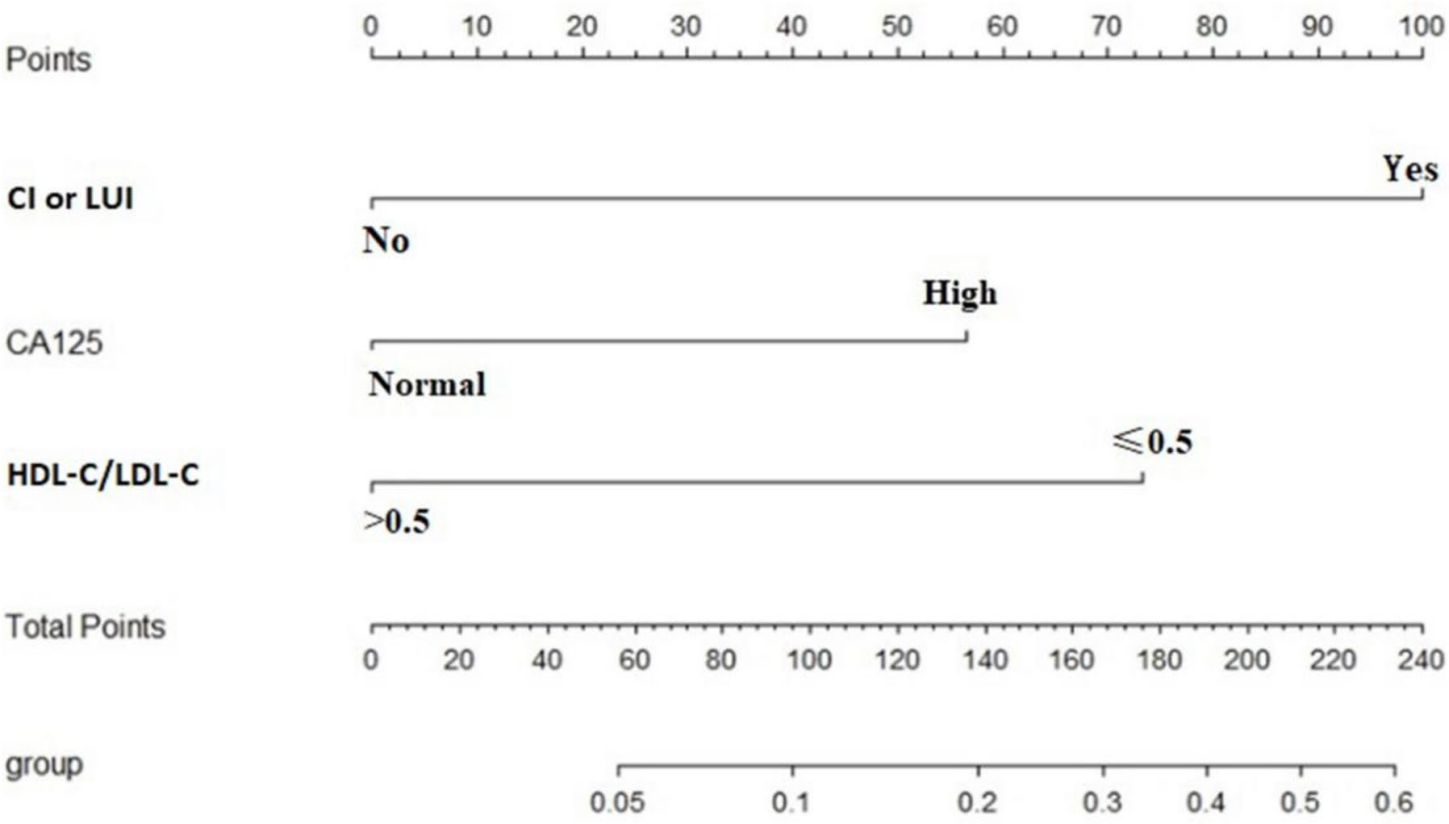
Nomogram predicting the probability of TP53mut in women with endometrial cancer. The nomogram prognostic model includes three variables: HDL-C/LDL-C, CA125 level, CI or LUI

The results of multivariate analysis of preoperative clinical parameters showed that HDL-C/LDL-C, CA125 level, and cervical or lower uterine involvement were independent influencing factors associated with TP53mut (*p* = 0.016, 0.047, < 0.001; Table 4). Based on logistic regression results, a nomogram prognostic model (TP53mut prognostic model, TPMM) was established as follows: TPMM = -1.385 × HDL-C/LDL-C + 1.068 × CA125 + 1.89 × CI or LUI. A nomogram was constructed based on the prediction model, as shown in Fig. 2.

**Table 4:** Multivariate logistic analysis of preoperative parameters for predicting TP53mut

	**β**	**OR (95% CI)**	***p***
**HDL-C/LDL-C (> 0.5)**	-1.385	0.250(0.081,0.771)	0.016
**CA125 (High)**	1.068	2.910(1.016,8.338)	0.047
**CI or LUI (Yes)**	1.890	6.619(2.344,18.696)	< 0.001

*Abbreviations*: *CI or LUI* Cervical involvement or lower uterine involvement according to imaging

### Diagnostic accuracy of TPMM for predicting TP53mut in the part A and model validation in the part B

**Fig. 3:**
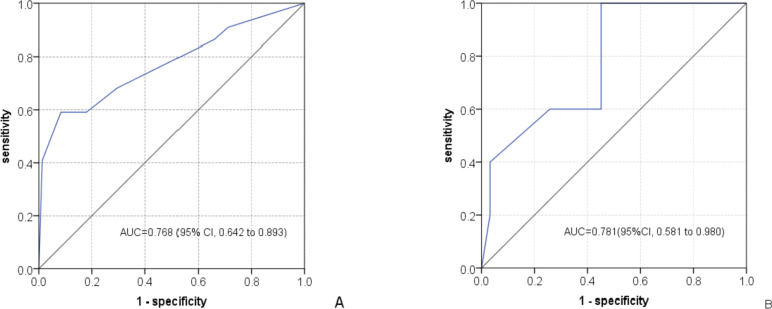
Receiver operating characteristic (ROC) curves of the constructed model for predicting TP53mut. **A **Model representation in the part A; **B **Model representation in the part B

The AUCs of TPMM for predicting TP53mut in the part A and part B were 0.768 (95% CI, 0.642 to 0.893) and 0.781 (95% CI, 0.581 to 0.980), respectively. The ROC curves were shown in Fig. 3. Diagnostic performance of TPMM for predicting TP53mut efficacy in part A and part B were shown in Table 5.

**Table 5:** Diagnostic performance of TPMM for predicting TP53mut efficacy in the part A and part B

	**AUC(95% CI)**	**Cutoff**	**Sen**	**Spe**	**PPV**	**NPV**	**Acu**
Part A	0.768(0.642 to 0.893)	≥ 2	13/22(59.1)	137/156(87.8)	13/32(40.6)	137/146(93.8)	150/178(84.3)
Part B	0.781(0.581 to 0.980)	/	2/5(40.0)	30/31(96.8)	2/3(66.7)	30/33(90.9)	32/36(88.9)

*Abbreviations*: *Sen* Sensitivity, *Spe* Specificity, *PPV* Positive predictive value, *NPV* Negative predictive value, *Acu* Accuracy

### Predicting clinical outcomes with TPMM in the part A

**Fig. 4:**
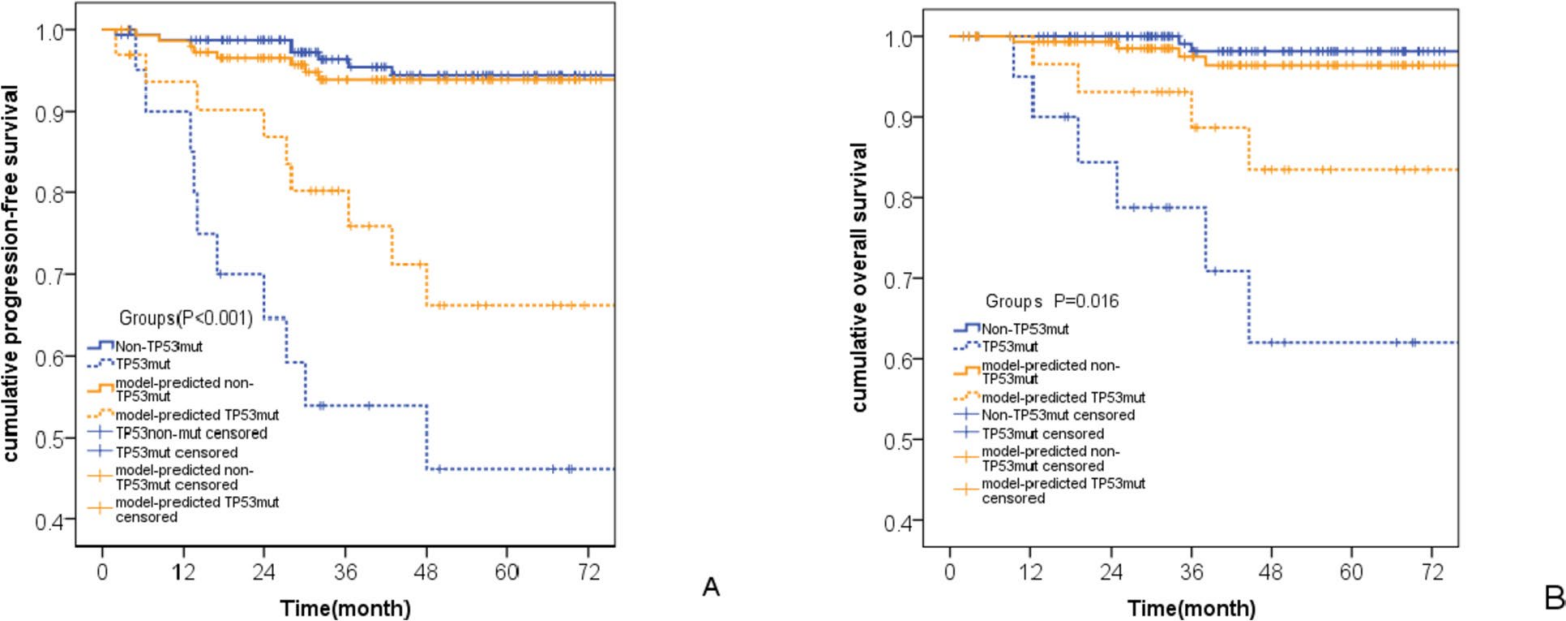
Comparison between TPMM predicted outcomes and actual outcomes in the part A. **A** Comparison of the prediction of progression-free survival; **B ** Comparison of the prediction of overall survival

The median follow-up time for the 178 patients was 47 months (95% CI, 43–55 months). A total of 18 patients (10.1%) relapsed, and 8 patients (4.5%) died. TP53mut patients had worse PFS and OS than non-TP53mut patients (both* p* < 0.001). The PFS and OS of TPMM-predicted TP53mut patients were also worse than those of TPMM-predicted non-TP53mut patients (*p* < 0.001, 0.016; Fig. 4).

## Discussion

Given the significant correlation between molecular typing and the prognosis of EC, molecular typing of EC is widely recommended in clinical practice guidelines. Since the survival outcomes of the TP53mut subtype among the four molecular subtypes are significantly worse than those of the other three subtypes, the effective identification of patients with the TP53mut subtype before surgery is particularly important for surgical decision-making and treatment. Therefore, in our study, the TP53mut subtype and three other molecular subtypes were dichotomized to screen out the characteristics of the preoperative clinical parameters associated with TP53mut, and a nomogram prediction model, TPMM, consisting of three preoperative parameter variables was constructed and verified.

In our study, the counts of neutrophils and monocytes in patients in the TP53mut group were significantly higher than those in patients in the non-TP53mut group. In fact, in recent years, many studies have supported the role of the systemic inflammatory response in the occurrence, progression and prognosis of tumors [15–17]. Peripheral blood cells, including neutrophils, lymphocytes and monocytes, are biomarkers of tumor immunity and play a crucial role in the systemic inflammatory response [18]. Various cytokines released by tumor tissues can stimulate the elevation of neutrophils, which provides a suitable microenvironment for the occurrence and development of tumors in turn. In patients with carcinoma, the functions of neutrophils are more varied. It can show defensive function and lead to the activation of some neutrophils to promote tumor stimulatory factors. In addition, the increase in neutrophils can also inhibit the activation of T lymphocytes, thus weakening the inhibition of tumor growth [19]. A large number of studies have also reported that monocytes have some protumor functions, such as differentiation into tumor-associated macrophages (TAMs), cytokine transfer, inhibition of T cells, promotion of angiogenesis and extracellular matrix remodeling [20]. Multiple factors are produced by TAMs, such as tumor growth factors and angiogenic factors, accelerating tumor progression and invasion [21]. Monocytes can also promote immune escape by limiting the infiltration of activated CD8 + T cells into the tumor microenvironment [22]. Therefore, an elevated monocyte count is a feature of tumor invasiveness. Additionally, monocytes are an independent predictor of poor prognosis of EC, which is associated with high-grade tumors, advanced disease, lymph node metastasis and positive ascites cytology [23, 24]. It has also been reported that the monocyte count is a useful biomarker for predicting tumor recurrence and progression [25]. Obviously, these studies suggest that elevated neutrophil and monocyte counts are associated with a poorer prognosis. This is consistent with the results of our study, in which TP53mut patients with the worst prognosis had significantly higher neutrophil and monocyte counts than non-TP53mut patients. This seems to explain the mechanism by which elevated neutrophil and monocyte counts are associated with poorer prognosis, possibly due to the association with TP53 mutations.

P53 is involved in regulating various metabolic pathways, including lipid metabolism [26], inhibiting lipid synthesis, and promoting lipid decomposition and fatty acid oxidation through various pathways [27]. P53 can inhibit the mevalonate pathway and reduce cholesterol synthesis. However, when P53 is mutated, it will lead to upregulation of the mevalonate pathway, increasing cholesterol synthesis and promoting the progression of liver cancer, pancreatic cancer and breast cancer [28–30]. Hydroxymethylglutaryl coA reductase is a key enzyme in the mevalonate pathway, and its inhibitors are statins. A large number of clinical studies have supported the antitumor effects of statins and other mevalonate pathway inhibitors [31, 32], including EC [33, 34]. Analysis of lipid metabolism in our study showed that the level of LDL-C in TP53mut patients was higher than that in non-TP53mut patients and further showed a lower ratio of HDL-C/LDL-C. LDL-C in the body is a kind of lipoprotein particle that carries cholesterol into peripheral tissue cells and can be oxidized into oxidized low-density lipoprotein (OX-LDL). When LDL-C, especially OX-LDL, is excessive, more cholesterol accumulates on the artery wall and causes atherosclerosis. Therefore, elevated levels of LDL-C are associated with an increased incidence of atherosclerosis, for which statins are effective drugs, and it seems to be understood that the mechanism by which statins improve the prognosis of EC involves reducing LDL-C levels thereby slowing the progression of EC. In addition, an association between LDL-C and non-endometrial EC was found in a Mendelian randomized study. The results of the study showed that the reduction in LDL-C was negatively correlated with the risk of non-endometrial-like EC [35]; in other words, the increase in LDL-C was positively correlated with the risk of non-endometrial-like carcinoma. The results of the study also showed that elevated LDL-C was associated with TP53 mutations, which were mostly non-endometrioid cancers, so our results were consistent with the results of that study.

As a commonly used tumor biomarker in clinical practice, an increase in CA125 is associated with poor prognosis of EC. Toole et al. reported that CA125 could be used as a preoperative risk stratification factor for lymph node metastasis of endometrioid cancer and was significantly correlated with lymph node metastasis [36]. Li et al. also found that the proportion of lymph node metastasis in EC patients with CA125 ≥ 35 U/ml was higher than that in patients with CA125 < 35 U/ml [37]. The results of our study showed that CA125 was higher in TP53mut patients, which may be due to the higher lymph node metastasis rate in patients with TP53mut (21/22, 95.5%).

Involvement of the cervix or lower uterine segment in endometrial carcinoma is associated with adverse prognostic factors, such as myometrial infiltration, serous surface of the uterus involvement, lymph node metastasis, and higher histological grade [38]. Cokmez et al. found that lower uterine involvement and lymphovascular space invasion were significantly correlated with lymph node metastasis [39]. Kizer et al. also showed that lower uterine segment involvement was associated with a high risk of recurrence and death in patients with early EC [40]. Although the above studies determined lower uterine segment or cervical involvement through postoperative pathology, a high proportion of positive patients could be identified through preoperative imaging examination. Yildirim et al. achieved 87.5%, 80% and 85% diagnostic accuracy of deep myometrial infiltration, lower uterine segment and cervical invasion by 3D vaginal ultrasound and higher diagnostic accuracy of MRI for lower uterine segment and cervical invasion [41]. In our study, the logistic regression analysis showed that cervical or inferior uterine involvement was one of the independent influencing factors associated with TP53mut by preoperative imaging.

Finally, we constructed a TP53mut prediction model (TPMM) with good diagnostic accuracy in validation population, and survival analysis showed the model can identify patients with different prognostic risk. To the best of our knowledge, this is the first study to construct a prediction model for TP53mut of EC with preoperative clinical parameters and validate it using both survival data and validation sample. The model is potentially useful for clinical practice as clinicians might acknowledge the TP53 mutation information at the preoperative time point without any invasive harm to patients.

However, there are some limitations to our study. First, our sample size was limited, and the validation sample was enrolled from the single center at a different period, which was actually not an independent validation set. Second, the robustness of the model may be influenced by potential factors such as long-term use of lipid-lowering drugs like statins, long-term use of the antiplatelet drug like aspirin. Recent infections could affect neutrophils, monocytes, and lymphocytes. Cervical involvement or lower uterine involvement in the model strongly depends on the experience of the radiologist for the accurate interpretation to the images. Third, we did not consider the factors of postoperative adjuvant therapy in the prognosis prediction, so the prediction results of the model were different from the real prognosis of patients. Furthermore, multi-center study with larger sample size should be initiated to test the effectiveness of the preoperative factors for predicting TP53mut in EC.

## Conclusions

We developed a highly predictive model, TPMM, with three preoperative noninvasive parameters for predicting the TP53mut subtype in EC. This model had good diagnostic accuracy, and survival analysis showed the model can identify patients with different prognostic risk. This can help gynecological oncologists plan the overall treatment of patients before surgery.

## Data Availability

The raw sequence data reported in this paper have been deposited in the Genome Sequence Archive (Genomics, Proteomics & Bioinformatics 2021) in National Genomics Data Center (Nucleic Acids Res 2022), China National Center for Bioinformation / Beijing Institute of Genomics, Chinese Academy of Sciences (Accession number: HRA004565) that are publicly accessible at https://ngdc.cncb.ac.cn/gsa/.

## Abbreviations

TP53mut: TP53 mutant
TCGA: The Cancer Genome Atlas
PFS: Progression-free survival
OS: Overall survival
NSMP: No specific molecular profile
NGS: Next-generation sequencing
FFPE: Formalin fixed, paraffin-embedded
TMB: Tumor mutation burden
MSI: Microsatellite instability
LN: Lymph node
EC: Endometrial cancer
LVSI: Lymphovascular space invasion
DMI: Deep myometrial infiltration
LNM: Lymph node metastasis
NCCN: The National Comprehensive Cancer Network
LVSI: Lymphovascular Invasion
TP: Total protein
Alb: Albumin
A/G: Albumin/globulin ratio
TCHO: Total cholesterol
TG: Triglycerides
HDL-C: High density lipoprotein cholesterol
LDL-C: Low density lipoprotein cholesterol
CI or LUI: Cervical involvement or lower uterine involvement according to imaging
OR: Odds Ratio
CI: Confidence Interval
ROC: Receiver Operating Characteristic
AUC: Area Under the Curve
C-index: Concordance index
BMI: Body Mass Index
TPMM: TP53mut prognostic model
Sen: Sensitivity
Spe: Specificity
PPV: Positive predictive value
NPV: Negative predictive value
Acu: Accuracy
TAMs: Tumor-associated macrophages
OX-LDL: Oxidized low-density lipoprotein
